# Supplementary material for: Cancer Immunotherapy with Lipid Nanoparticles Loaded with a Stimulator of Interferon Genes Agonist against Renal Tumor Lung Metastasis
Source: Pharmaceutics. 2023 Dec 26;16(1):31. doi: 10.3390/pharmaceutics16010031 (PMC10819482; doi:10.3390/pharmaceutics16010031)
Supplement: Supplementary file 1 [file pharmaceutics-16-00031-s001.zip › pharmaceutics-2763810-supplementary.pdf]

Supplementary data

Cancer immunotherapy with lipid nanoparticles loaded with a STING agonist against renal tumor lung metastasis.

Takashi Nakamura*, Shun Sasaki, Yusuke Sato, Hideyoshi Harashima*

Faculty of Pharmaceutical Sciences, Hokkaido University, Kita-12, Nishi-6, Kita-ku, Sapporo 060-0812, Japan

*Correspondence:
Takashi Nakamura
Faculty of Pharmaceutical Sciences, Hokkaido University
Sapporo, Hokkaido 060-0812, Japan.
Telephone: +81-11-706-3735, Fax: +81-11-706-3734
E-mail: tnakam@pharm.hokudai.ac.jp

Hideyoshi Harashima
Faculty of Pharmaceutical Sciences, Hokkaido University
Sapporo, Hokkaido 060-0812, Japan.
Telephone: +81-11-706-2197, Fax: +81-11-706-2197
E-mail: harasima@pharm.hokudai.ac.jp

Table S1. Sequences of the primer pairs used for the RT-qPCR

Gene	Forward primer	Reverse primer
Cd3	ATGCGGTGGAACACTTTCTGG	GCACGTCAACTCTACACTGGT
Cd4	CTTCGCAGTTTGATCGTTTTGAT	CCGGACTGAAGGTCACTTTGA
Cd8	AAGAAAATGGACGCCGAACTT	AAGCCATATAGACAACGAAGGTG
Foxp3	TGCAGGGCAGCTAGGTACTTG	TCGGAGATCCCCTTTGTCTTATC
Ctla4	CATGGTGTCGCCAGCTTTC	GGTAATCTAGGAAGCCCACTGTA
Lag3	GGCCTCGATGATTGCTAGTCC	CGGTGAGTTGTAGACAGGCA
Nkg2d	GCACTAACTACCAGTCAACCTG	CTCGAACAACGAACATTGGAGA
Ifng	ATGAACGCTACACACTGCATC	CCATCCTTTTGCCAGTTCCTC
Cd11c	CTGGATAGCCTTTCTTCTGCTG	GCACACTGTGTCCGAACTCA
Xcr1	CAGAGTCAGATGCTCTCAGTATCC	GGACAATGGTAGAGATGGTGGA
Ccl5	TGCTCCAATCTTGCAGTCGT	GGCTAGGACTAGAGCAAGCAA
Gusb	GTGGTATGAACGGGAAGCAAT	AACTGCATAATAATGGGCACTGT

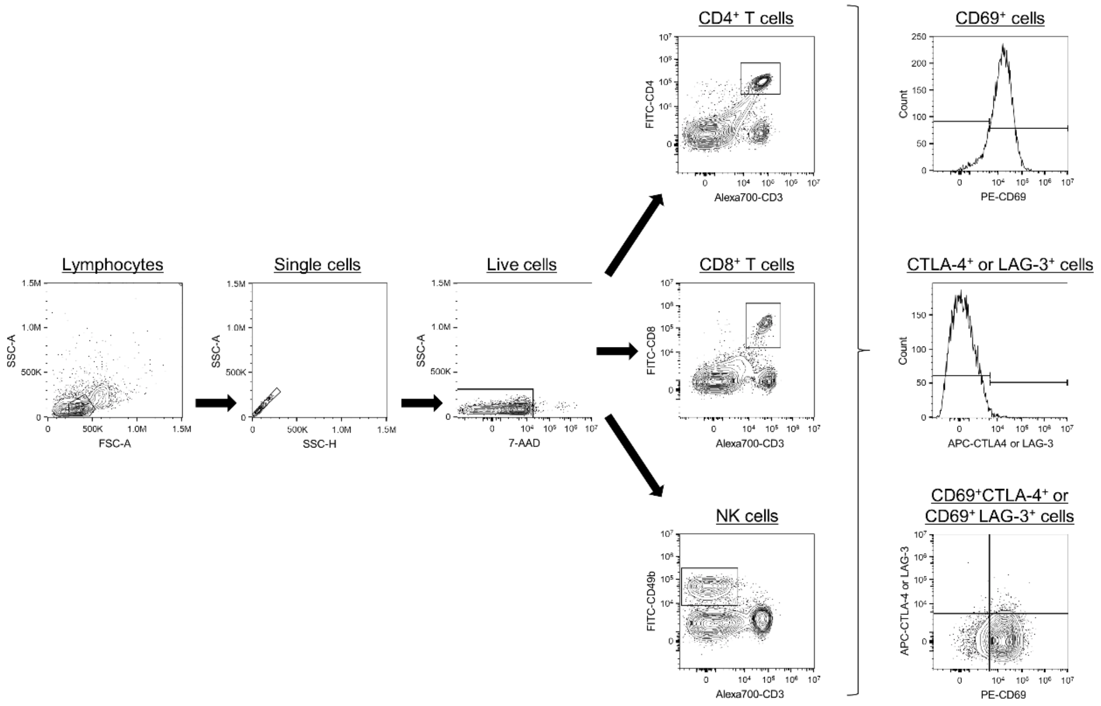

Figure S1. Gating strategy for the FCM analysis of lungs with Renca metastasis.

After gating the lymphocytes, single cells and gated live cells, $CD4^+$ T cells ($CD3^+CD4^+$ cells), $CD8^+$ T cells ($CD3^+CD8^+$ cells), NK cells ($CD3^-CD49b^+$ cells) were identified. In each immune cell population, the $CD69^+$ gate, the CTLA-4^+ gate, the LAG-3^+ gate, the $CD69^+$CTLA-4^+ gate, and the $CD69^+$LAG-3^+ gate was set by using isotype controls.
